# Isolation and identification of pathogenic fungi and oomycetes associated with beans and cowpea root diseases in Oman

**DOI:** 10.7717/peerj.6064

**Published:** 2018-12-13

**Authors:** Asma Al-Jaradi, Issa Al-Mahmooli, Rhonda Janke, Sajeewa Maharachchikumbura, Nadiya Al-Saady, Abdullah M. Al-Sadi

**Affiliations:** 1 Oman Botanic Garden, Diwan of Royal Court, Al-Khod, Muscat, Oman; 2 Department of Crop Sciences, College of Agricultural and Marine Sciences, Sultan Qaboos University, Al-Khod, Muscat, Oman; 3 Oman Animal and Plant Genetic Resources Center, Muscat, Oman

**Keywords:** *Phaseolus*, Pothogenicity, Disease diagnosis, Aggressiveness, Pathogens

## Abstract

The purpose of this study was to characterize fungal and oomycete species associated with root diseases of *Phaseolus vulgaris*, *Vigna unguiculata*, *V. radiata* and* Vicia faba* in Oman. Root samples were collected from plants suffering from weakened growth and yellowing symptoms. Fungal species were isolated on 2.5% potato dextrose agar amended with 10 mg l^−1^ rifampicin and 200 mg l^−1^ of ampicillin and identification was based on sequences of the internal transcribed spacer region of the ribosomal RNA gene (ITS rRNA), glycerol-3-phosphate dehydrogenase (GPDH), translation elongation factor-1 alpha (TEF), beta-tubulin (TUB), calmodulin (CMD), actin (ACT). Isolations yielded 204 fungal isolates belonging to nine different genera, with most isolates belonging to *Alternaria* and *Fusarium*. Molecular identification revealed that the isolates belong to 20 fungal species, the most dominant of which was *Alternaria alternata*. Pathogenicity tests were conducted on each plant species. The inoculations on *P. vulgaris* revealed that *Pythium aphanidermatum* induced rotting, damping-off and wilt symptoms while *Fusarium equiseti* induced yellowing symptoms on the leaves. *Rhizoctonia solani* produced lesions and root rot on *Vigna unguiculata* while *Curvularia muehlenbeckiae* and *Curvularia caricae-papayae* produced root lesions on the roots of *V. unguiculata* and *V. radiata*, respectively. *Alternaria alternata* produced brown symptoms on the tap root of *Vicia faba*. *P. aphanidermatum* resulted in a significant reduction in the fresh weight, dry weight and shoot length of *Phaseolus vulgaris*. The study shows that several fungal species can be found associated with the roots of beans and *Vigna unguiculata* in Oman and can result in varying disease symptoms. This is the first report of root lesions produced by *Curvularia muehlenbeckiae* on the roots of *V. unguiculata* and by *C. caricae-papayae* on *V. radiata* worldwide.

## Introduction

In Oman, several legume crops such as *Phaseolus vulgaris* L. (common bean) *Vigna unguiculata* L. (cowpea), *V. radiata* L. (mung bean), *Vicia faba* L. (Fababean), *Pisum sativum* L. (Pea), *Cajanus cajan* L. (pigeon pea) and others are grown in different regions. The area cultivated in Oman in 2013 with *Vigna unguiculata*, *Phaseolus vulgaris*, *Vicia faba* and *Vigna radiata* was over 3337 ha, 396 ha, 194 ha and 37 ha, respectively. Al Dakiliya and Al Batinah are among the major places for growing these legumes (MAF, 2013). However, these legumes are also grown in most of the agricultural regions in the country, including Al Dhahira, Al Buraimi, Al Sharqiya and Dhofar.

Several fungal genera have been reported to associate with foliar and root diseases on beans, peas and other legumes. These include *Alternaria*, *Aphanomyces*, *Aspergillus*, *Curvularia*, *Drechslera*, *Fusarium*, *Penicillium*, *Rhizoctonia* and *Rhizopus* species which have been detected on *P. vulgaris*, *Vicia faba*, *Vigna unguiculata* and *Pisum sativum* (Abdulwehab, El-Nagerabi & Elshafie, 2015; Gossen et al., 2016; Sousa et al., 2017; Spedaletti et al., 2017; Zeng et al., 2017). Vural & Soylu (2012) reported root and foliage infection of kidney bean with soil-borne fungal pathogens including *Fusarium solani* (Mart.) Sacc., *F. oxysporum* Schltdl., *Rhizoctonia solani* J.G. Kühn, *Macrophomina phaseolina* (Tassi) Goid., *Pythium ultimum* Trow, *Sclerotinia sclerotiorum* (Lib.) de Bary, *Sclerotium rolfsii* Sacc. and *Alternaria* spp*.* Infection of roots by soilborne pathogens can affect water and nutrient uptake (Peña et al., 2013). Generally, fungal root disease epidemics on plants are influenced by several factors such as soil moisture, soil fertility, and the occurrence of other diseases and pests (Belete, Ayalew & Ahmed, 2013; Degraeve et al., 2016; Li et al., 2016; Naseri & Ansari Hamadani, 2017). In Oman, little attention has been given to root diseases of beans and related crops, which makes the establishment of management strategies to these diseases a challenge (Moghal et al., 1993).

The present study designed to identify the most common fungal pathogens associated with root diseases in *Phaseolus vulgaris*, *Vigna unguiculata*, *V. radiata* and *Vicia faba*. Specific objectives include: (1) to identify fungal species associated with root diseases of the four crops using morphological and molecular tools; and (2) to test pathogenicity of the most common fungal species. Knowledge about the pathogens involved in root rot of beans and cowpea will help design strategies to manage the disease in these crops.

## Materials and Methods

### Survey and collection of samples

A survey was carried out in four geographical locations in Oman: Al Batinah North, Al Batinah South, Al Dakiliya and Al Sharqiya South. During the survey, root samples were collected from *Phaseolus vulgaris* (eight fields), *Vigna unguiculata* (seven fields), *Vigna radiata* (1 field) and *Vicia faba* (five fields). 10 samples were randomly collected walking in a zig-zag pattern over each field in sterile plastic bags from 2–3 months old plants showing wilt or weak growth. The collection of samples was during August 2015 to March 2016. The samples were transported to the Plant Pathology Research Laboratory at Sultan Qaboos University in a cooler box within 12 hrs. of the collection of samples.

### Isolation of fungi

Fungi were isolated from the collected root samples. First, samples were washed with tap water to remove soil debris, and then immersed in 1% sodium hypochlorite (NaOCl) for 1–2 min. Samples were transferred to sterile distilled water (SDW) for 2–3 min followed by drying on sterile filter paper. The root were cut to small 2–3 mm pieces and four pieces were placed on 2.5% Potato Dextrose Agar (PDA) medium amended with 10 mg l^−1^ rifampicin and 200 mg l^−1^ of ampicillin. The plates were kept at room temperature in the laboratory for 7 to 10 days. Fungal growth on each plate were sub-cultured to a new plate and the plates were kept in an incubator at 27–29 °C. The growth of fungi was observed daily.

### Identification of fungi

The isolated fungi were first identified morphologically using a compound microscope. The fungal isolates were then initially identified using sequences of the internal transcribed spacer region of the ribosomal RNA (ITS rRNA). Additional loci were amplified and sequenced, namely: glycerol-3-phosphate dehydrogenase (GPDH) for *Curvularia*, actin (ACT) for *Macrophomina*; beta-tubulin (TUB) for *Aspergillus*, *Macrophomina*, *Penicillium* and *Talaromyces;* calmodulin (CMD) for *Aspergillus*, *Macrophomina*, *Penicillium* and *Talaromyces;* and translation elongation factor (TEF) for *Fusarium* and *Macrophomina.* Fungal cultures were harvested by scraping mycelia from 10-day old PDA plates into a sterile eppendorf tube and kept at −80 °C. DNA extraction steps for fungal isolates were done as described by Al-Sadi et al. (2015), with some modifications. The fungal mycelium and spores were ground in a mortar and pestle with sand and then 600 µl of lysis buffer was added. The mixtures were mixed and incubated at 65 °C for 1 hr. After that, 600 µl of phenol: chloroform: isoamylalchohol (25:24:1) was added, vortexed and centrifuged for 15 min at 10,000 g. About 300 µl of supernatant was transferred to new eppendorf tubes and 10µl of sodium acetate and 180 µl of isopropanol were added. The mixture was incubated at −20 °C overnight. The mixtures were centrifuged for 2 min at 10,000 g and the supernatant was discarded. The pellet was washed using 600 µl of 70% ethanol, followed by centrifugation for 2 min at 10,000 g. Then, the pellets were dried for 10–15 min. The pellets were suspended in 100 µl of autoclaved sterile distilled water and DNA concentrations of samples were measured with a spectrophotometer NanoDrop 1000 (Thermo Fisher Scientific, Waltham, MA, USA) and stored at −20 °C until used.

### Polymerase chain reaction and sequencing

Polymerase Chain Reaction (PCR) was run as described by Al-Sadi et al. (2015), using PuRe Taq Ready-To-Go™ PCR beads, 1 µl from each primer (0.4 mM), 25–50 ng of DNA, and sterile distilled water up to 25 µl. ITS region was run according to the conditions described by Al-Sadi et al. (2015). The primers EF-728F and EF-986R were used for amplification of TEF (O’Donnell & Cigelnik, 1997); ACT region using primers ACT 512F and ACT 783R (Carbone & Kohn, 1999); TUB region using primers BT2A and BT2B (Glass & Donaldson, 1995); CMD using primers CMD and CMD6 (Hong et al., 2005); GPDH region with primers gpd1 and gpd2 (Berbee, Pirseyedi & Hubbard, 1999). The thermal cycler programs were as Woudenberg et al. (2013), Samson et al. (2014) and Sarr et al. (2014). PCR samples were cleaned and sequenced at MACROGEN, Korea.

### Pathogenicity test

Pathogenicity of the most commonly isolated fungi during the study or known pathogenic species based on literature were tested on the four hosts: *Phaseolus vulgaris*, *Vigna unguiculata*, *Vigna radiata*, and *Vicia faba*. Only healthy seeds with uniform size were chosen for the pathogenicity test. Peat moss and trays were autoclaved twice at 121 °C for 30 min (Nerey et al., 2010). Then, the seeds were sown in the autoclaved soil peat moss and the trays were kept in a growth chamber at 30 °C, 70% humidity and 16-h photoperiod.

Five mm diameter discs obtained from 3–14 day old fungal cultures were placed at the hypocotyl area of each seedling (Nerey et al., 2010). Seedlings were inoculated after producing 2–4 true leaves and seven days after inoculation, disease symptoms were recorded. Plants inoculated with agar plugs served as controls. Seedlings were watered every 2 days, maintained in greenhouse under 30 °C and no fertilizer added during the experiment. The experimental set-up was designed with five seedlings per treatment and the experiment was repeated twice. The percentage of seedlings developing wilt symptoms, development of necrotic areas/rotting on the roots, stem length, root length, fresh weight, and dry weight were recorded for each seedling. Root and shoot length was measured using a ruler while for dry weight, the plants were dried in an oven at 80 °C for 24 hrs (Ali et al., 2008). Re-isolations were established from the seedlings developing disease symptoms as explained previously.

### Data analysis

For phylogenetic analysis, the two complimentary sequences for each isolate were aligned and edited using Chromas (v. 1.41). To show the species relationship within *Aspergillus, Penicillium*, *Talaromyces*, *Pythium*, *Rhizoctonia*, *Macrophomina*, *Curvularia* and *Cladosporium*, separate phylogenetic trees were prepared. Multiple sequence alignments were generated with MEGA v. 6 (Tamura et al., 2013). A maximum likelihood analysis was performed using raxmlGUI v. 1.3 (Silvestro & Michalak, 2012). The optimal ML tree search was conducted with 1000 separate runs, using the default algorithm of the program from a random starting tree for each run. The gaps were treated as missing data. The final tree was selected among suboptimal trees from each run by comparing likelihood scores under the GTR+GAMMA substitution model. Sequences derived in this study were deposited in GenBank (Table 1).

**Table 1:** Fungal species and their accession numbers.

Species name	Isolate No:	Host	Location	GenBank accesion					
				ITS	GPDH	TUB	CMD	TEF	ACT
*Alternaria alternata*	SQU 14008	*Vigna unguiculata*	Al Suwaiq	KY684261	MG979062	−	−	−	−
*Alternaria alternata*	SQU 14010	*Phaseolus vulgaris*	Sohar	KY684262	−	−	−	−	−
*Alternaria alternata*	SQU 14012	*Phaseolus vulgaris*	Sohar	KY684263	−	−	−	−	−
*Alternaria alternata*	SQU 14115	*Vigna unguiculata*	Al Suwaiq	KY684264	MG979061	−	−	−	−
*Alternaria alternata*	SQU 14116	*Vicia faba*	Al Rustaq	KY684265	MG979063	−	−	−	−
*Aspergillus flavus*	SQU 14000	*Phaseolus vulgaris*	Sohar	KY684266	−	MH000345	MH000358	−	−
*Aspergillus quadrilineatus*	SQU 14043	*Phaseolus vulgaris*	Sohar	KY684269	−	MH000344	MH000357	−	−
*Aspergillus terreus*	SQU 14026	*Phaseolus radiatus*	Sohar	KY684268	−	MH000347	MH000360	−	−
*Aspergillus terreus*	SQU 14072	*Phaseolus vulgaris*	Barka	KY684270	−	MH000346	MH000359	−	−
*Cladosporium perangustum*	SQU 14028	*Vigna radiata*	Sohar	KY684271	−	−	−	−	−
*Curvularia hawaiiensis*	SQU 14161	*Vigna unquiculata*	Al Musanah	MH025387	MG979057	−	−	−	−
*Curvularia hawaiiensis*	SQU 14037	*Vigna unquiculata*	Al Musanah	MH025388	MG979060	−	−	−	−
*Curvularia muehlenbeckiae*	SQU 14002	*Vigna unquiculata*	Al Musanah	KY684272	MG979059	−	−	−	−
*Curvularia caricae-papayae*	SQU 14058	*Phaseolus vulgaris*	Sohar	KY684275	−	−	−	−	−
*Curvularia caricae-papayae*	SQU 14036	*Phaseolus radiatus*	Sohar	KY684274	−	−	−	−	−
*Curvularia caricae-papayae*	SQU 14005	*Phaseolus vulgaris*	Sohar	KY684273	MG979055	−	−	−	−
*Curvularia caricae-papayae*	SQU 14056	*Phaseolus vulgaris*	Sohar	MH025389	MG979058	−	−	−	−
*Fusarium equiseti*	SQU 14017	*Phaseolus vulgaris*	Sohar	KY684278	−	−	−	MH000352	−
*Fusarium solani*	SQU 14015	*Phaseolus vulgaris*	Sohar	KY684277	−	−	−	MH000353	−
*Fusarium* sp*.*	SQU 14019	*Vigna radiata*	Sohar	−	−	−	−	MH000355	−
*Macrophomina phaseolina*	SQU 14035	*Vigna unquiculata*	Al Hamra	KY684279	−	MH000343	MH000356	MH000354	MH000342
*Penicillium canescens*	SQU 14069	*Vicia faba*	Nakhal	KY684281	−	MH000349	MH000362	−	−
*Penicillium glabrum*	SQU 14001	*Phaseolus radiatus*	Sohar	KY684280	−	MH000348	MH000361	−	−
*Pythium aphanidermatum*	SQU 14003	*Phaseolus vulgaris*	Barka	KY684285	−	−	−	−	−
*Pythium aphanidermatum*	SQU 14004	*Phaseolus vulgaris*	Sohar	KY684286	−	−	−	−	−
*Pythium aphanidermatum*	SQU 14222	*Phaseolus vulgaris*	Barka	KY684284	−	−	−	−	−
*Pythium aphanidermatum*	SQU 14067	*Vigna unquiculata*	Al Musanah	MH025375	−	−	−	−	−
*Pythium spinosum*	SQU 14022	*Vicia faba*	Al Hamra	KY684282	−	−	−	−	−
*Pythium spinosum*	SQU 14064	*Vicia faba*	Al Hamra	KY684283	−	−	−	−	−
*Rhizoctonia* sp.	SQU 14046	*Vicia faba*	Al Hamra	MH025383	−	−	−	−	−
*Rhizoctonia* sp.	SQU 14050	*Phaseolus vulgaris*	Barka	MH025384	−	−	−	−	−
*Rhizoctonia* sp.	SQU 14034	*Phaseolus vulgaris*	Barka	MH025385	−	−	−	−	−
*Rhizoctonia* sp.	SQU 14021	*Phaseolus vulgaris*	Barka	MH025386	−	−	−	−	−
*Rhizoctonia solani*	SQU 14129	* Vigna unquiculata*	Al Sharqiya South	KY684287	−	−	−	−	−
*Rhizoctonia solani*	SQU 14135	* Vigna unquiculata*	Al Musanah	KY684288	−	−	−	−	−
*Rhizoctonia solani*	SQU 14057	* Vigna unquiculata*	Al Suwaiq	MH025376	−	−	−	−	−
*Rhizoctonia solani*	SQU 14018	* Vigna unquiculata*	Al Suwaiq	MH025377	−	−	−	−	−
*Rhizoctonia solani*	SQU 14088	* Vigna unquiculata*	Al Musanah	MH025378	−	−	−	−	−
*Rhizoctonia solani*	SQU 14081	*Vicia faba*	Al Hamra	MH025379	−	−	−	−	−
*Rhizoctonia solani*	SQU 14147	*Vicia faba*	Al Hamra	MH025381	−	−	−	−	−
*Rhizoctonia solani*	SQU 14062	*Phaseolus vulgaris*	Al Suwaiq	MH025382	−	−	−	−	−
*Talaromyces purpureogenus*	SQU 14107	* Vigna unquiculata*	Al Musanah	KY684290	−	MH000350	MH000363	−	−

The differences between treatments in dry weight, fresh weight, stem length, root length and disease incidence were analysed using Statistical Analysis Software program (SAS) at *P* < 0.05 using GLM analysis (ANOVA model) (DiMaggio, 2013). Separation of means was based on Tukey’s Studentized range test (SAS).

## Results

### Survey

**Figure 1:**
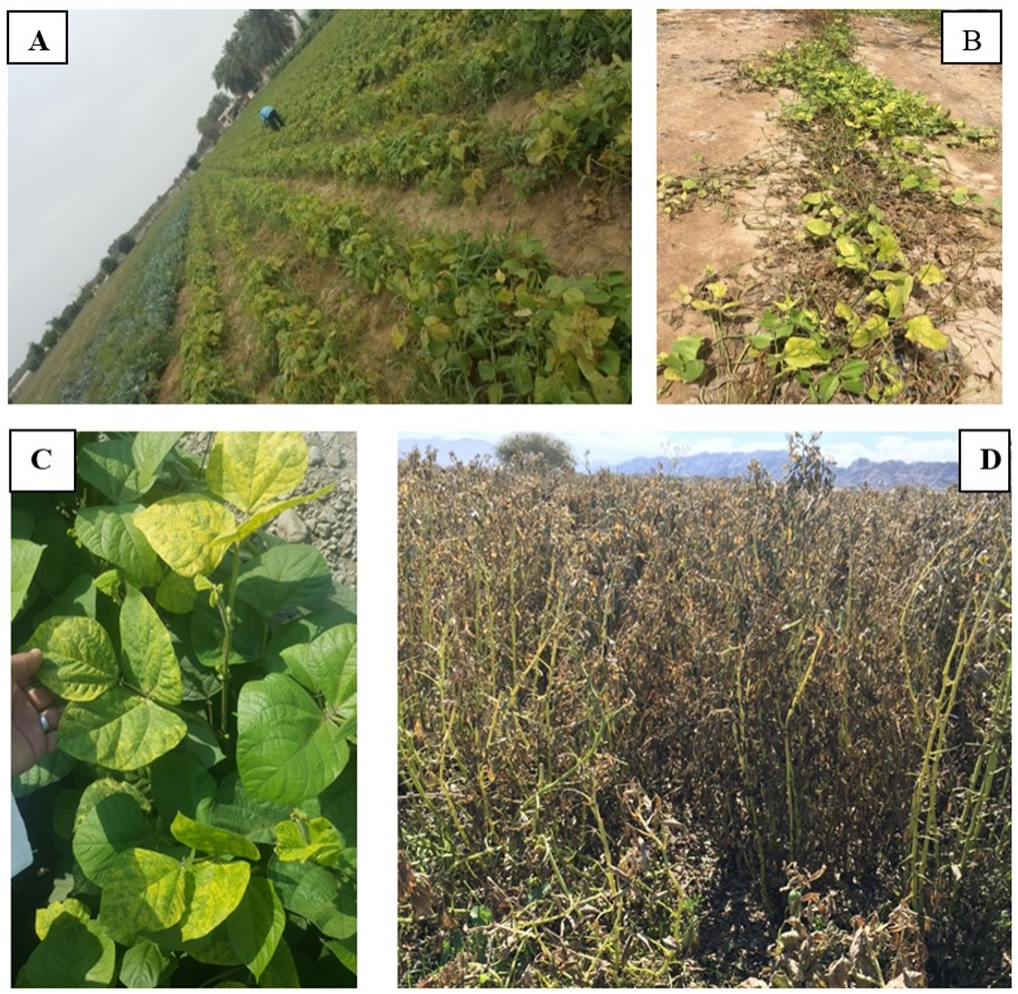
Fields of *Phaseolus vulgaris* (A) *Vigna unguiculata* (B) *V. radiata* (C) and *Vicia faba* (D) from which root samples were collected. Photos taken by Asma Al-Jaradi.

During the survey, a total of 237 root samples belonging to different species of beans and cowpeas were collected (Table 2). The survey in Oman showed that *Phaseolus vulgaris*, *Vigna unguiculata*, *V. radiata* and *Vicia faba* suffer from early wilt symptoms, including weakened growth and yellowing. Approximately 5–10% of the visited fields were found to suffer from these symptoms. No severe wilt symptoms were observed in any of the fields which were visited (Fig. 1).

**Table 2:** Species of beans and cowpea collected from different locations in Oman.

Common name	Scientific name	Location	Sample size
Common bean	*Phaseolus vulgaris* L.	Al Batinah North	28
		Al Batinah South	63
Cowpeas	*Vigna unguiculata* L*.*	Al Batinah North	15
		Al Batinah South	29
		Al Sharqiya South	29
		Al Dakiliya	10
Mung beans	*Vigna radiata* L.	Al Batinah North	12
Fababean	*Vicia faba* L*.*	Al Batinah South	23
	* *	Al Dakiliya	28

### Identification of fungi

We isolated 204 fungal isolates and initially identified using morphological traits following Aoki & O’Donnell (1999), Manamgoda et al. (2012), Woudenberg et al. (2013), Samson et al. (2014) and Sarr et al. (2014). Sporulating isolates belonged to eight genera; *Fusarium*, *Macrophomina*, *Alternaria*, *Curvularia*, *Cladosporium*, *Aspergillus*, *Penicillium* and *Talaromyces*. Phylogenetic and sequence analyses showed that our samples belong to ten different genera namely; *Pythium*, *Fusarium*, *Rhizoctonia*, *Macrophomina*, *Alternaria*, *Curvularia*, *Cladosporium*, *Aspergillus*, *Penicillium* and *Talaromyces*. The most dominant genus was *Alternaria* (Table 3).

**Table 3:** Frequency (%) of recovery of fungal species from roots of *Phaseolus*, *Vigna* and *Vicia* cultivars following molecular identification.

Taxon	*Phaseolus vulgaris*	*Vigna unguiculata*	*V. radiata*	*Vicia faba*	Distribution of fungi
*Alternaria alternata*	44	0	13^a^	33^a^	Al Batinah & Al Dakhliya
*Aspergillus flavus*	3	7	0	0	Al Batinah
*Aspergillus quadrilineatus*	3	0	0	0	Al Batinah
*Aspergillus* sp*.*	0	0	13	0	Al Batinah
*Aspergillus terreus*	3	7	25	0	Al Batinah & Al Dakhliya
*Cladosporium perangustum*	0	0	13	0	Al Batinah
*Curvularia hawaiiensis*	0	14^a^	0	0	Al Batinah
*Curvularia muehlenbeckiae*	0	7^a^	0	0	Al Batinah
*Curvularia caricae-papayae*	9	0	13^a^	0	Al Batinah
*Fusarium equiseti*	3^a^	0	0	0	Al Batinah
*Fusarium solani*	3	0	0	0	Al Batinah
*Fusarium* sp*.*	12	0	13	0	Al Batinah
*Macrophomina phaseolina*	0	7	0	0	Al Dakhliya
*Penicillium canescens*	0	0	0	11	Al Batinah
*Penicillium glabrum*	0	0	13	0	Al Batinah
*Pythium aphanidermatum*	9^a^	7	0	0	Al Batinah
*Pythium spinosum*	0	0	0	22^a^	Al Dakhliya
*Rhizoctonia solani*	3	36^a^	0	22	Al Batinah, Al Dakhliya & Al Sharqiya
*Rhizoctonia* spp*.*	9	0	0	11	Al Batinah & Al Dakhliya
*Talaromyces purpureogenus*	0	14	0	0	Al Batinah

**Notes.**

a Fungal species and their host used in pathogenicity test.

**Figure 2:**
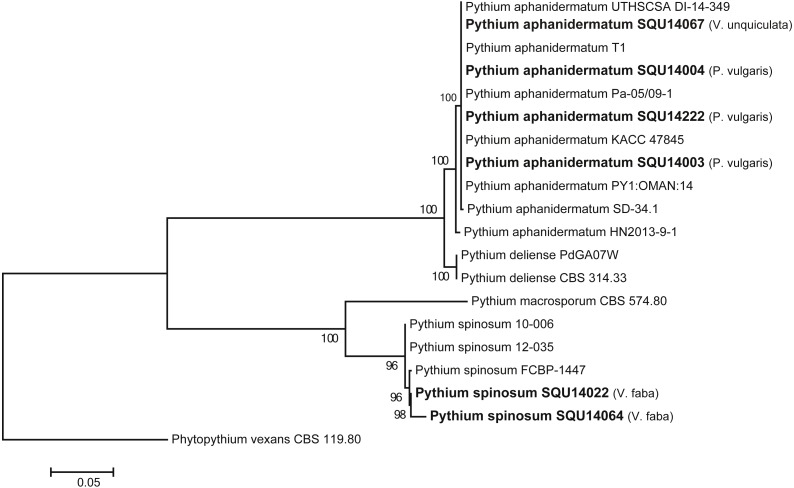
Phylogram generated from maximum likelihood analysis based on ITS sequence data of *P. aphanidermatum*, *P. spinosum* and related species. Isolates derived from this study are in bold. RAxML bootstrap support values above 50 are given at the nodes. The tree is rooted with *Phytopythium vexans*.**

The ITS tree analyses consisted with 20 taxa including six *Pythium* isolates obtained during this study and *Phytopythium vexans* (CBS 119.80) as the out-group taxon (Fig. 2). The dataset consisted of 758 characters including gaps. The *Pythium* isolates obtained in this study clustered with two previously published species, namely, *P. aphanidermatum* (four isolates) and *P. spinosum* (two isolates) with 100% ML support. The *P. aphanidermatum* was isolated from *Phaseolus vulgaris* from Barka and Sohar and one isolate on *Vigna unguiculata* from Al Musanah. Two isolates of *P. spinosum* were isolated from *Vicia faba* in Al Hamra.

The *Fusarium* strains isolated during this study cannot be assigned with confidence to any particular species on the basis of ITS sequences as megablast searches in GenBank and blast searches in FUSARIUM-ID database were 100% identical to several species. The closest hits using a megablast search of the TEF sequence of SQU 14015 which was isolated from *P. vulgaris* from Sohar was 100% identical to the *Fusarium solani* isolates TOR-397 (242/242; GenBank KT716212), FRC S761 (242/242; GenBank DQ247348). With use of the FUSARIUM-ID database, the closest species on TEF belong to *F. solani* species complex (NRRL22938, NRRL22938, NRRL31164, NRRL31164) with (100%) identity. The TEF sequences of the *Fusarium* isolates obtained from *P. vulgaris* from Sohar 237/237(100%) and 237/237(100%) similar to *F. equiseti* isolate DAOM213327 (GenBank DQ842087) and FusB11 (GenBank GQ160449), respectively. With use of the FUSARIUM-ID database, the closest species on TEF belong to *F. incarnatum-equiseti* species complex (NRRL34070, NRRL32864, NRRL36548, NRRL32522) with (100%) identity. Moreover, some isolates were obtained from *P. vulgaris* and *Vigna radiata* but were not assigned to any species of *Fusarium* (Table 3).

**Figure 3:**
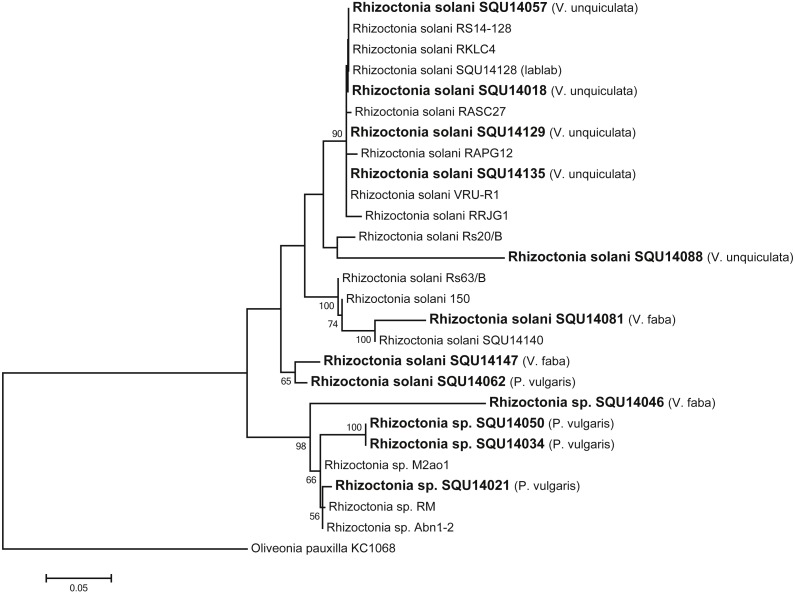
Phylogram generated from maximum likelihood analysis based on ITS sequence data of *Rhizoctonia* species. Isolates derived from this study are in bold. RAxML bootstrap support values above 50 are given at the nodes. The tree is rooted with *Oliveonia pauxilla*.

*Rhizoctonia* dataset contained 27 taxa including 14 isolates of *Rhizoctonia* isolates obtained during this study from different hosts and a total of 788 characters including gaps. Based on the phylogenetic analysis of ITS sequence data, 10 isolates were found to belong to *Rhizoctonia solani* and four isolates were kept as *Rhizoctonia* spp. as they could not assigned to any of the known species of *Rhizoctonia* (Fig. 3).

**Figure 4:**
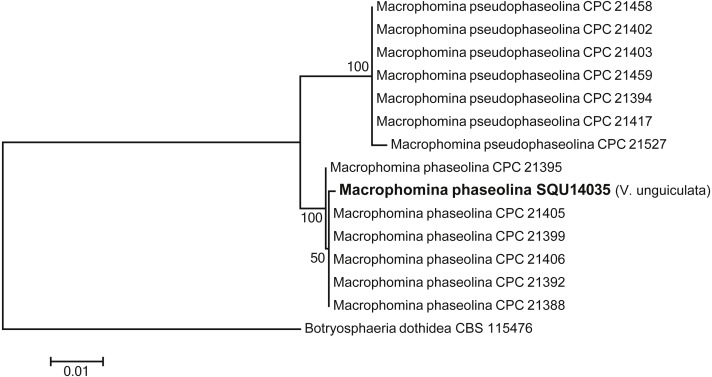
Phylogram generated from maximum likelihood analysis based on combined ITS, TEF, TUB, CMD, ACT sequence data of *Machrophomina* species. Isolates derived from this study are in bold. RAxML bootstrap support values above 50 are given at the nodes. The tree is rooted with *Botryosphaeria dothidea*.

One isolate of *Macrophomina* was isolated during this study. Based on the phylogenetic analysis of combined ITS, TEF, TUB, CMD and ACT sequence data, the isolate was clustered with known *M. phaseolina* isolates with 100% ML support*.* The alignment contained 15 taxa including *Botryosphaeria dothidea* as the out-group taxon and a total of 2210 characters including gaps. *Macrophomina phaseolina* isolate was obtained from *V. unguiculata* from Al Hamra (Fig. 4).

Twenty nine *Alternaria* isolates were obtained during this study and they were identical in conidial morphology. Out of twenty nine, five isolates were sequenced using ITS and GPDH gene regions (Table 1). However, based on ITS megablast searches they cannot be assigned with confidence to any particular species as they were 100% identical to several species. BLAST searches of the GPDH sequences of *Alternaria* isolates showed 100% similarity with *Alternaria alternata* isolates in the GenBank. Details of the hosts of the *Alternaria* isolates are shown in Table 3.

**Figure 5:**
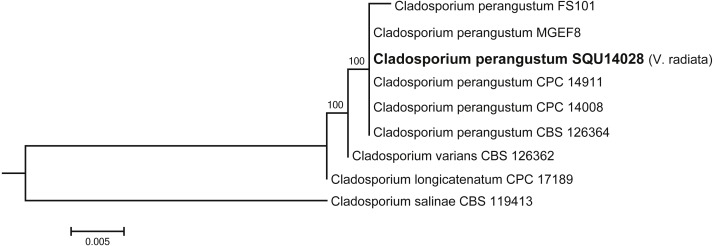
Phylogram generated from maximum likelihood analysis based on ITS sequence data of *Cladosporium* species. Isolates derived from this study are in bold. RAxML bootstrap support values above 50 are given at the nodes. The tree is rooted with *Cladosporium salinae*.

*Cladosporium* dataset contained nine taxa including one isolates obtained during this study from *V. radiata* (SQU 14028) and *Cladosporium salinae* (CBS 119413) as the out-group taxon. The alignment contained total of 506 characters including gaps. In the phylogenetic tree SQU 14028, clustered together with the five isolates of *Cladosporium perangustum* with 100% bootstrap supports (Fig. 5).

**Figure 6:**
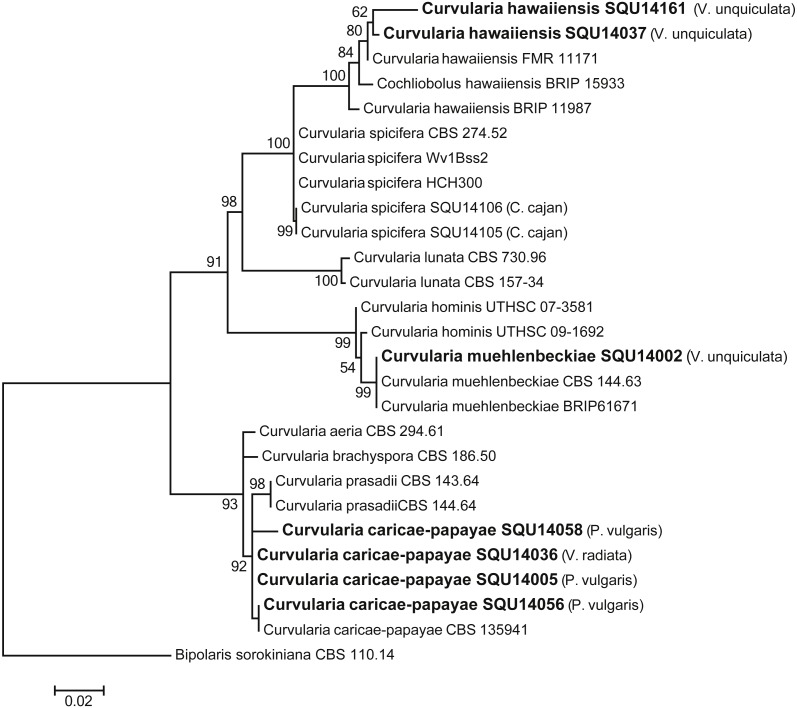
Phylogram generated from maximum likelihood analysis based on combined ITS and GPDH sequence data of *Curvularia* species. Isolates derived from this study are in bold. RAxML bootstrap support values above 50 are given at the nodes. The tree is rooted with *Bipolaris sorokiniana*.

The analysed *Curvularia* dataset consisted of combined ITS and GPDH sequence data (a total of 1137 characters including gaps) for seven isolates of *Curvularia* isolated during this study with *Bipolaris sorokiniana* (CBS 110.14) as the outgroup taxon. The seven *Curvularia* isolates clustered with three previously published species, namely, *C. c aricae-papayae* (four isolate)*, C. hawaiiensis* (two isolates), and *C. muehlenbeckiae* (one isolates) (Fig. 6).

**Figure 7:**
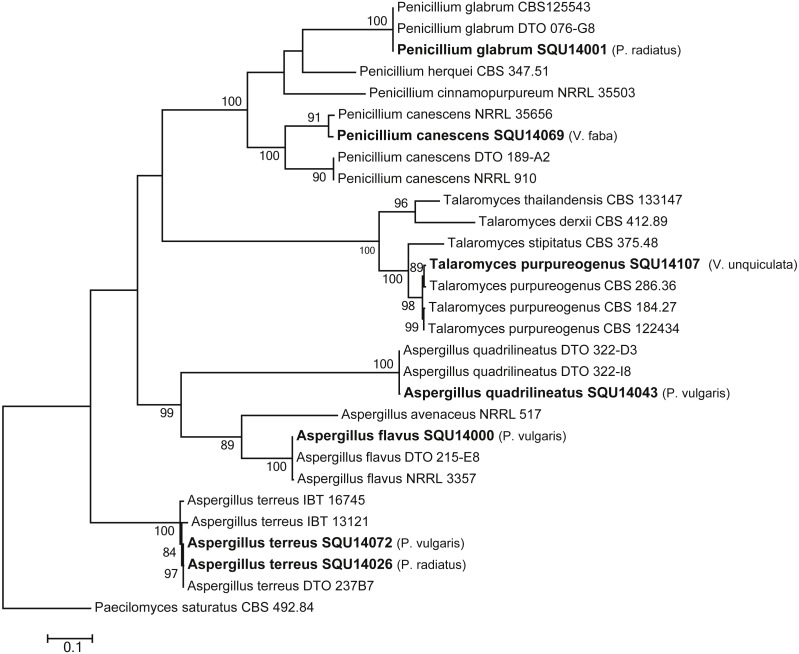
Phylogram generated from maximum likelihood analysis based on combined ITS, CMD and TUB sequence data of *Aspergillus, Penicillium* and *Talaromyces* species. Isolates derived from this study are in bold. RAxML bootstrap support values above 50 are given at the nodes. The tree is rooted with *Paecilomyces saturatus*.

The phylogenetic analyses were carried out using combined ITS, CMD and TUB sequence data from taxa of *Aspergillus*, *Penicillium* and *Talaromyces* (Fig. 7). The final multiple alignment data comprised 29 taxa and 1804 characters including gaps and the species were identified as *A. flavus*, *A. terreus*, *A. quadrilineatus*, *Penicillium canescens*, *P. glabrum* and *Talaromyces purpureogenus.* These isolates were obtained from different hosts from different regions in Oman (Table 3).

### Pathogenicity test on *Phaseolus vulgaris*

**Figure 8:**
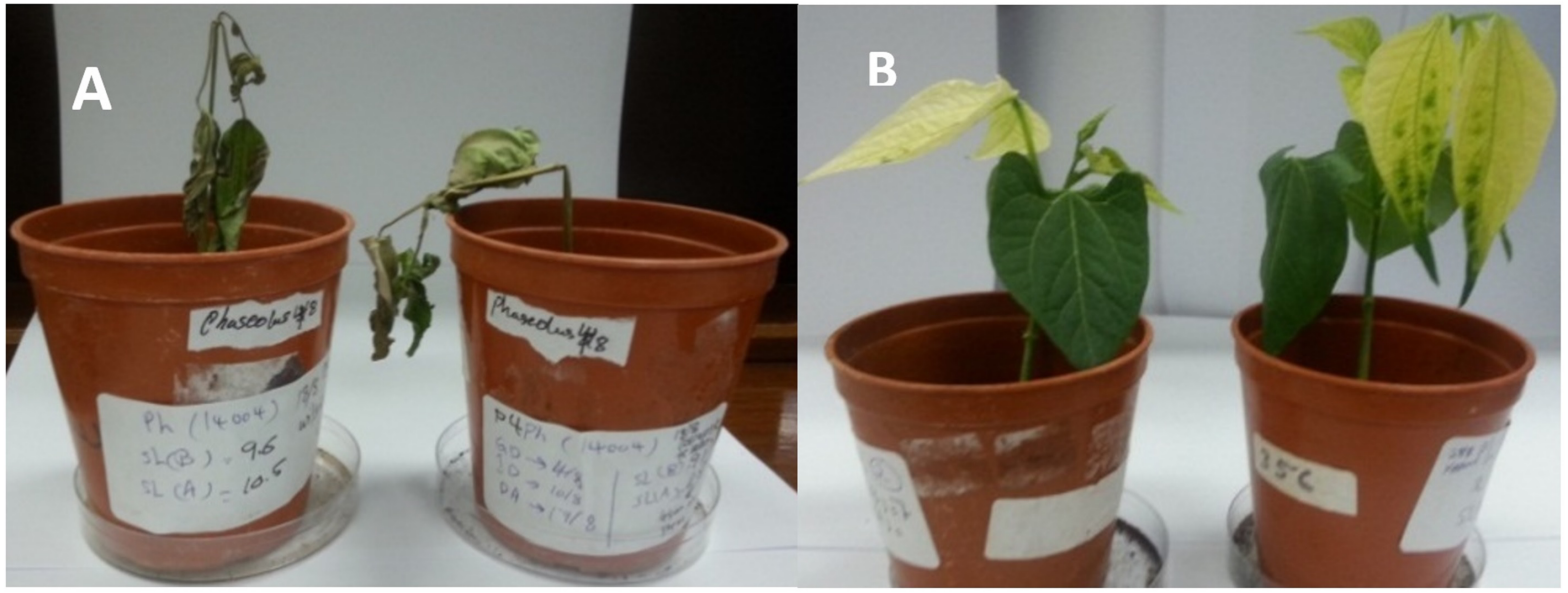
Wilt (A) and yellowing (B) symptoms on *Phaseolus vulgaris* inoculated with *Pythium aphanidermatum* and *Fusarium equiseti*, respectively. Photos taken by Asma Al-Jaradi.

**Figure 9:**
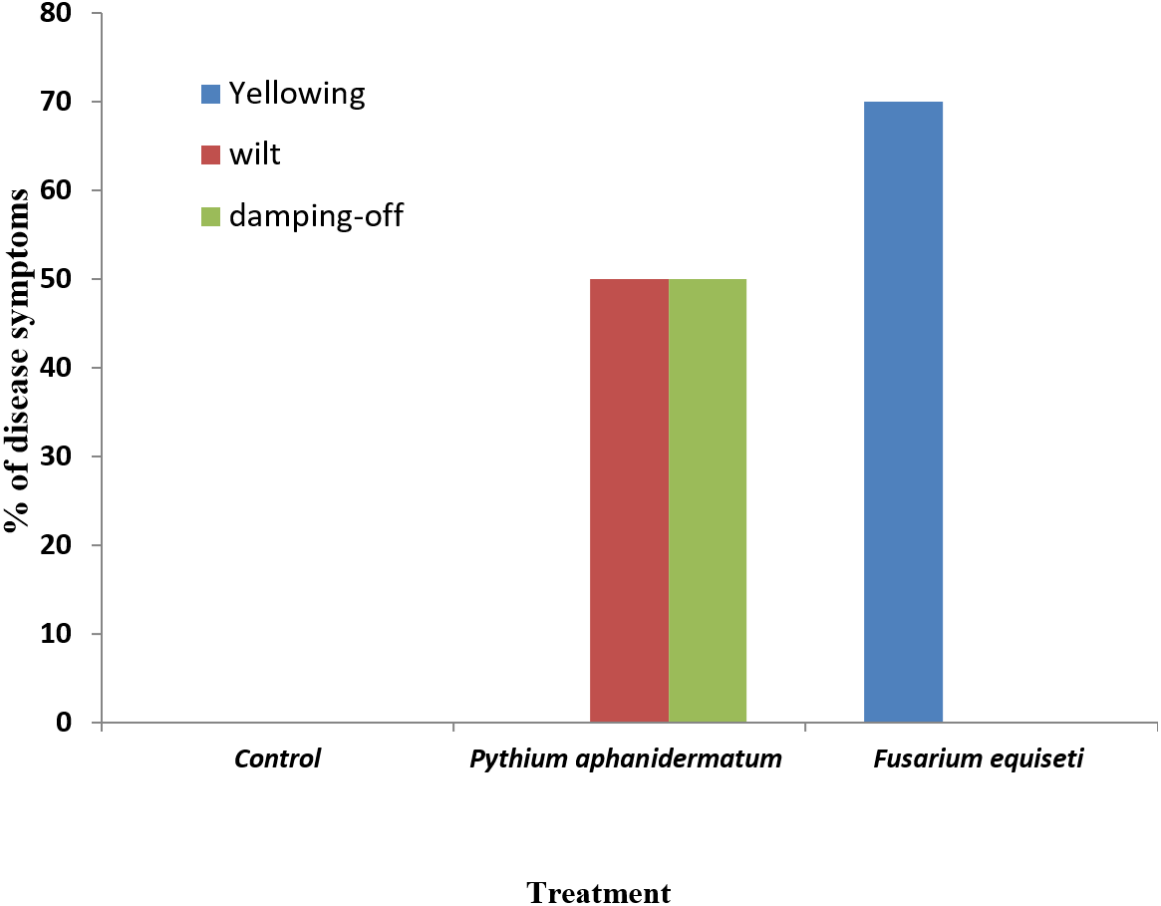
Incidence of damping-off, wilt and yellowing symptoms on *Phaseolus vulgaris* following inoculation with *Pythium aphanidermatum* and *Fusarium equiseti.* Columns with the same letters in the same category are not significantly different from each other (*P* < 0.05; Tukey’s Studentized range test, SAS).

Inoculation of *Phaseolus vulgaris* seedlings with *Pythium aphanidermatum* resulted in the production of damping-off and wilt symptoms in 50% of the seedlings (Figs. 8A &  9). The inoculated plants showed brown lesions and root rot symptoms on the hypocotyl and main root and significant reductions in the fresh weight, dry weight and stem length compared to the control (*P* < 0.05) (Table 4).

**Table 4:** Effect of fungal inoculation on the fresh weight, dry weight and stem length on 10 tested plants.

Plant name	Treatment	Fresh weight	Dry weight	Stem length	
*Phaseolus vulgaris*	Control	2.58 a	0.305 a	4.85 a	
	*Pythium aphanidermatum*	1.12 b	0.205 b	1.55 b	
	*Fusarium equiseti*	2.93 a	0.395 a	5.57 a	
					
*Vigna unguiculata*	Control	3.16 a	0.330 a	9.69 ab	
	*Curvularia muehlenbeckiae*	3.27 a	0.345 a	9.00 a	
	*C. hawaiiensis*	1.32 a	0.155 a	9.78 ab	
	*Rhizoctonia solani*	2.42 a	0.280 a	9.27 b	
					
*Vigna radiata*	Control	0.56 a	0.050 a	7.89 a	
	*Curvularia caricae-papaya*	0.48 a	0.085 a	9.54 a	
	*Alternaria alternata*	0.90 a	0.095 a	8.38 a	
					
*Vicia faba*	Control	1.50 a	0.175 a	10.74 a	
	*Pythium spinosum*	1.29 a	0.165 a	8.93 a	
	*Alternaria alternata*	1.38 a	0.205 a	10.33 a	

**Notes.**

Means with the same letter in the same column for the same plant species are not significantly different from each other (Tukey’s Studentized Range test, SAS).

Inoculation with *Fusarium equiseti* resulted in yellowing symptoms in 70% of the inoculated seedlings and resulted in discoloration on the tap roots (Figs. 8B and 9). However, there was no significant effect of *F. equiseti* on the fresh weight, dry weight and stem length of the inoculated seedlings (*P* > 0.05; Table 4). Control seedlings remained symptomless.

### Pathogenicity test on *Vigna unguiculata*

Inoculation of *Vigna unguiculata* with *Rhizoctonia solani* resulted in the production of lesions and root rot symptoms on the hypocotyl, taproot and secondary roots of 20%, 60% and 20% of the seedlings, respectively (data not presented). The mean lesion length were 0.1 cm (SD =  ± 0.03), 1.6 cm (SD =  ± 0.1) and 1.1 cm (SD =  ± 0.1) on the hypocotyl, taproot and secondary roots, respectively. There was no significant effect of *R. solani* on the fresh weight, dry weight and shoot length of *V. unguiculata* (Table 4). *Curvularia muehlenbeckiae* induced discoloration only on 20% of the secondary roots, while *C. hawaiiensis* caused no symptoms or significant reductions on the fresh weight, dry weight or stem length of *V. unguiculata* seedlings . Control seedlings remained symptomless.

### Pathogenicity test on *Vigna radiata*

Inoculation of *Vigna radiata* with *Curvularia caricae-papayae* showed that it induced brown lesions on the tap root and secondary roots, but without having a significant effect on the fresh weight, dry weight or stem length of seedlings. *Alternaria alternata* did not produce any symptoms and did not have any significant effect on the fresh weight, dry weight or shoot length of seedlings (Table 4). Control seedlings did not show any symptoms.

### Pathogenicity test on *Vicia faba*

*Vicia faba* seedlings inoculated with *Alternaria alternata* showed brown symptoms on the tap root, 1.4 cm in length. However, *Pythium spinosum* did not induce any symptoms on the inoculated seedlings. Both fungi did not have any significant effects on the fresh weight, dry weight or shoot length of *V. faba* seedlings (*P* > 0.05) (Table 4).

## Discussion

Our survey showed that several fungal species are associated with the root of beans and cowpeas. *Pythium aphanidermatum* has been detected on *Phaseolus vulgaris*. In the pathogenicity test, *P. aphanidermatum* caused wilting and damping-off symptoms and brown lesions and root rot on hypocotyl and taproot of treated plants. However, *P. spinosum* did not cause any symptoms in *Vicia faba*. *Pythium* species have been reported to cause several diseases on beans including root and hypocotyl rot on *Phaseolus vulgaris* (Kokalis-Burelle et al., 2017; Li et al., 2016; Nerey et al., 2010). *Pythium* causes several disease symptoms such as pre-emergence and post-emergence damping-off, blight, stunting and rotting on seeds, stems and roots (Hall, 1991; Nzungize et al., 2012). However, there were no disease symptoms on *Vicia faba* by *P. spinosum*. This could be because *P. spinosum* prefers low temperatures <25 °C (Al-Sa’di et al., 2007).

We detected *Fusarium equiseti* on *Phaseolus vulgaris*. In the pathogenicity test, treated *P. vulgaris* showed yellowing symptoms with presence of lesions and root rot on the taproot. However, the pathogen did not affect the fresh weight, dry weight or shoot length. *Fusarium* has been isolated from *Phaseolus vulgaris*, *Vicia faba*, *Vigna unguiculata* and *Pisum sativum* (Abdulwehab, El-Nagerabi & Elshafie, 2015). *F. equiseti* is one of the causal agents of foot and root rot disease which infects *Phaseolus vulgaris*, *Pisum sativum* and other crops (Clarkson, 1978). The pathogen was also detected in the seeds of *P. vulgaris* (Abdulwehab, El-Nagerabi & Elshafie, 2015).

We isolated *Rhizoctonia solani* from *Vigna unguiculata* in Oman. This pathogen caused lesions and root rot symptoms on the hypocotyl, taproot and secondary roots of *V. unguiculata* inoculated with *R. solani* but with a non-significant reduction in fresh and dry weight, stem and root length. *R. solani* is the causal agent of Rhizoctonia root rot on *Phaseolus vulgaris* and other beans (El-Mohamedy et al., 2017; Peña et al., 2013). It is reported as the causal agent of web blight disease on *Vigna unguiculata* (*Onesirosan, 1975*). It causes damping-off, root and hypocotyl rot, brown lesions on hypocotyl and roots (Nerey et al., 2010; Peña et al., 2013) and crown blight, fruit rot and leaf blight in *Phaseolus vulgaris* (Peña et al., 2013). Other disease symptoms are plant stunting and premature death (Hall, 1991).

*Alternaria alternata* resulted in lesions and root rot symptoms on the taproot of *Vicia faba*. *Alternaria* has been detected on different *Vicia faba*, *Pisum sativum* and *Vigna unguiculata* (Abdulwehab, El-Nagerabi & Elshafie, 2015). However, *Alternaria* spp. are usually known to be foliar pathogens, causing various types of spots and blights. This may explain why they were less pathogenic on the roots compared to other fungi.

In this study, three different *Curvularia* species were identified on different legumes; *C.  hawaiiensis* on *Vigna unguiculata* and *C. muehlenbeckiae* on *Phaseolus vulgaris*, *Vigna unguiculata* and *C. caricae-papayae on Vigna radiata*. In pathogenicity test, *C. muehlenbeckiae* caused lesions and root rot on the secondary roots of *Vigna unguiculata* and *C.  caricae-papayae* caused lesions on the taproot and secondary roots of *Vigna radiata*. *C. muehlenbeckiae* is a recently identified new species which was isolated from *Muehlenbeckia* sp. leaf in India and human chest in USA (Madrid et al., 2014). No previous studies have reported the association of *C. muehlenbeckiae* with root diseases on *Vigna unguiculata*.

In the present study, *Macrophomina phaseolina* has been detected on *Vigna unguiculata*. It has already been reported on *Phaseolus vulgaris* and demonstrated to cause root and hypocotyl rot disease alone or in complex with *Pythium* spp., *Rhizoctonia* spp. and *Fusarium* spp. (Bareja, Kumar & Lodha, 2010; Nerey et al., 2010).

Other fungal species were detected in this study, but no pathogenicity test was conducted from these fungi either because of their low recovery rate or because they are known to be saprophytes on roots. *C. perangustum* was detected on *Vigna radiata*. *Cladosporium* spp. can be isolated from various sources as saprobes (Bensch et al., 2012). They also responsible for foliar fungal disease and can survive well in the soil on plant debris for next season (Kraft & Pfleger, 2001). In this study, three *Aspergillus* species have been found on some of the collected samples; A. flavus on *Phaseolus vulgaris* and *Vigna unguiculata*, *A. terreus* on *P. vulgaris*, *Vigna unguiculata* and *Vigna radiata*, and *A. quadrilineatus* on *P. vulgaris*. Moreover, *Penicillium canescens* was detected on *Vicia faba* and *P. glabrum* on *Vigna radiata*. *Talaromyces purpureogenus* has been isolated in this study from *Vigna unguiculata*. Several species of *Talaromyces* are biocontrol agents against several plant diseases (Li et al., 2014; Yuan et al., 2017).

## Conclusions

Our study showed that *P. aphanidermatum* is the most aggressive pathogen compared to other pathogens, as it induced mortality within a short period. Although other pathogens should also not be neglected, the use of proper fungicides to control *P. aphanidermatum*, especially at early stages of growth, is important. This is the first report of *C. muehlenbeckiae* on *Vigna unguiculata* and *C. caricae-papayae* on *Vigna radiata*. However, this study does not eliminate the possibility of development of disease symptoms in these crops by any of the other fungal species, as pathogenicity is affected by several factors including the growth stage, environmental conditions, aggressiveness of the fungal isolate and time from inoculation to the development of symptoms. Future studies should investigate pathogenicity for fungal species with a low rate of recovery from plants.

##  Supplemental Information

10.7717/peerj.6064/supp-1 Data S1 Raw data

## Additional Information and Declarations

### DNA Deposition

The following information was supplied regarding the deposition of DNA sequences: DNA sequencing data are provided in Table 1.

### Data Availability

The following information was supplied regarding data availability: Sequences are available in GenBank under the accession numbers described in Table 1 and the raw data is available as Data S1.
